# Production of monodisperse polyurea microcapsules using microfluidics

**DOI:** 10.1038/s41598-019-54512-4

**Published:** 2019-11-29

**Authors:** Michael F. Thorne, Felix Simkovic, Anna G. Slater

**Affiliations:** 1 Department of Chemistry and Materials Innovation Factory, University of Liverpool, Crown Street, Liverpool, L69 7ZD UK; 2 Institute of Integrative Biology, University of Liverpool, Liverpool, L69 7ZB UK

**Keywords:** Polymer synthesis, Process chemistry

## Abstract

Methods to make microcapsules – used in a broad range of healthcare and energy applications – currently suffer from poor size control, limiting the establishment of size/property relationships. Here, we use microfluidics to produce monodisperse polyurea microcapsules (PUMC) with a limonene core. Using varied flow rates and a commercial glass chip, we produce capsules with mean diameters of 27, 30, 32, 34, and 35 µm, achieving narrow capsule size distributions of ±2 µm for each size. We describe an automated method of sizing droplets as they are produced using video recording and custom Python code. The sustainable generation of such size-controlled PUMCs, potential replacements for commercial encapsulated systems, will allow new insights into the effect of particle size on performance.

## Introduction

Microcapsules – that is, sub-mm size capsules with a solid shell and a solid or liquid core – have diverse applications across sustainability and energy, in healthcare, and in consumer products^1–9^. For example, microcapsules have been used for self-healing anticorrosion coatings^10,11^, energy storage materials^12,13^, and in catalysis^14^. By using encapsulation technology, manufacturers and researchers are able to use much smaller quantities of expensive or harmful ingredients^15,16^, or achieve a controlled release of the liquid core on response to a stimulus^17–19^. Despite the utility of polymer microcapsules, there are considerable environmental concerns regarding their persistence in the environment^20^ or use of harmful additives such as formaldehyde^21,22^. As the properties of microcapsules – such as release profile, permeability, and stability over time – often depend on particle size^23–25^, there is a strong drive to produce monodisperse microcapsules such that robust size/property relationships can be established. However, commonly used industrial methods of microcapsule production result in polydisperse populations, limiting the information that can be gained concerning the effect of size on their properties. A sustainable method of generating monodisperse, size-controlled polymer microcapsules is therefore highly desirable for research and development into the next generation of environmentally benign microcapsules.

**Figure 1:**
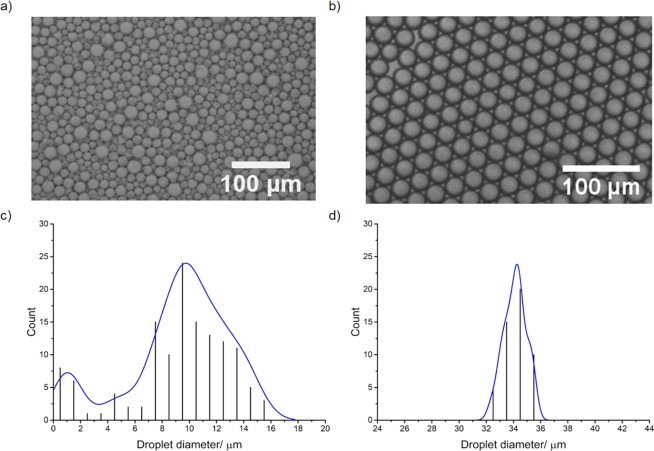
Size distribution of emulsion droplets produced by standard and microfluidic methods (**a**,**b**) Optical microscope images of o/w emulsions produced by (**a**) homogenizer and (**b**) microfluidic chip (Q_c_ = 100 µL min^−1^, Q_d_ = 5 µL min^−1^); (**c**,**d**) histograms of droplet size distribution from (**a**,**b**) analysed by ImageJ for (**c**) homogenized and (**d**) microfluidic chip produced droplets (Q_c_ = 100 µL min^−1^, Q_d_ = 5 µL min^−1^).

The most common method of polymer microcapsule production is interfacial polymerisation (IFP) at the interface of an oil-in-water (o/w) or water-in-oil (w/o) emulsion produced by high-shear mixing with a homogenizer^24^. First, a stable emulsion must be produced with the required droplet diameter; the droplet will form the core of the microcapsule. Polymerization occurs only at the boundary of the emulsion, ensuring that a thin film is formed around the droplet template. The size regime of the droplets produced is chiefly dependent on the emulsification device, surfactants present, and the energy applied to the system^26^; standard batch high-shear mixing methodologies result in poor control over the size distribution of the droplets (Fig. 1a), and hence the polymer microcapsules formed.

Microfluidic methods^27^ – that is, where reagents are flowed through micrometre-sized channels and mixed at a junction – are, by contrast, capable of extremely precise control over both droplet size and dispersity^28–30^. At the point of mixing, a high shear force is generated between the two immiscible fluids, resulting in droplet formation^29,31^. The shear force can be adjusted by altering the relative flow rates of the two input streams, which, along with channel size, controls the size of the resultant droplet. Thus, it is possible to continuously produce monodisperse emulsions of a desired size. Examples of such droplet production methods have been used in the synthesis of multifunctional magnetoresponsive microcapsules^32^, core-shell organosilicon capsules^33^, and biopolymer hydrogels^34^. Monodisperse polymer microcapsules of between 10–50 microns in diameter are of particular interest to the personal care industry^35^ to avoid the potential hazards of nanoparticles; microfluidic methods can readily access this size regime. Furthermore, microfluidic methods require very small amounts of material, and are sustainable due to the short reaction times, low energy usage, waste minimization, and energy and cost efficiency achievable with a continuous process^36,37^.

The choice of polymer, oil, and surfactant have a significant effect on the kinetics of polymerisation and the resultant microcapsule shell thickness^24,38–40^. Here, we also consider sustainability of the raw materials and the resultant polymer capsules. The ideal polymer shell from an industrial perspective is stable over the shelf-life of the product, capable of payload delivery at the required moment or rate, and not hazardous to the environment. Polyurea microcapsules (PUMCs) have been suggested as candidate materials that fulfil these criteria^38,41^. In terms of feedstocks, the oil used should ideally be from a cheap, renewable source, and function as an active ingredient in a product formulation. Limonene, commonly used in fragrance and foods, can be used as both a template and a payload, and is a sustainable by-product of the citrus industry found in peel^42^. Although polydisperse limonene microcapsules have been previously prepared^43–45^, there are no studies using microfluidic chips to generate limonene-containing microcapsules.

In this research, we use a microfluidic chip to generate monodisperse emulsion microdroplets of limonene containing diisocyanate monomer in an aqueous carrier fluid containing sodium dodecyl sulfate (SDS) and NaCl. We compare the size and polydispersity of the resultant droplets to samples produced by homogenization methods. By systematically varying the flow rate of the oil, we generate emulsions of tunable droplet diameter. Droplet formation is monitored *in situ*; the droplet size and polydispersity is measured from both still images and videos, the latter using an automated method. Methods of video processing of droplets using Labview have been previously reported^46,47^; here we use Python code for ease of accessibility. Interfacial polymerisation is achieved offline by collecting the droplets in a stirred solution of aqueous polyamine, which reacts with the diisocyanate to form a polyurea shell (see SI for reaction scheme). The resultant microcapsules are characterised by optical microscopy, SEM, and fluorescence microscopy, and found to have narrow size dispersity, high stability in air over at least 24 h, and the ability to carry a fluorescent payload. Having developed a sustainable method to produce size-controlled microcapsules on demand, we now seek to exploit this to understand the effect of size and dispersity on the performance of microcapsules in product formulations.

## Methods

### Batch synthesis

An aqueous solution of SDS and NaCl (1.0 wt. % and 1.5 wt. % respectively in 200 ml) and a solution of methylene diisocyanate (MDI) in limonene (0.3 wt. %, 10 ml) were prepared, mixed and homogenised at 8000 RPM for 2 minutes using an ULTRA-TURRAX T-25 homogeniser. The use of SDS and NaCl in these quantities resulted in the formation of emulsion droplets that were stable for at least 24 h. To form capsules, the resulting emulsion was allowed to stand for 10 minutes before a portion (1 mL) was injected into an aqueous solution of tetraethylenepentamine (TEPA), SDS, and NaCl (3.0 wt. %, 1.0 wt. %, and 1.5 wt. % respectively in 10 mL) and stirred at 100 RPM with a magnetic flea for 15 minutes. Capsules were left unstirred for 24 hours before being isolated via pipette and dried in air on a glass slide for imaging and SEM analysis.

### Microfluidic setup, droplet and capsule synthesis

A Dolomite system equipped with 2 Mitos compressed air pumps was used to generate flow rates of between 1–100 µL min^−1^. MDI dispersed in limonene (0.3 wt. %) and an aqueous solution of SDS and NaCl (1.0 and 1.5 wt. % respectively) were delivered to a Dolomite glass 2-reagent droplet chip with a junction size of 50 µm to generate monodisperse emulsion droplets (see SI for full details). The flow rate of the dispersed oil phase, Q_d_, was varied; the flow rate of the continuous water phase, Q_c_, was kept constant at 100 µL min^−1^. To avoid the potential for blockages, polymerisation was accomplished offline. Droplets were collected in a stirred (100 rpm, magnetic flea) solution of TEPA, SDS, and NaCl in water (3.0, 1.0, and 1.5 wt. % respectively in 10 mL). Droplets were collected for 15 minutes, after which time stirring was stopped and the solution left undisturbed for 24 h prior to being collected via pipette and dried in air on a glass slide for analysis.

### Characterisation of droplets and microcapsules

Droplets were imaged at the junction with a high speed optical microscope capable of capturing both still images and videos. Samples of emulsion and microcapsules were collected before and after polymerisation and imaged using offline optical microscopy. Typically, droplets and microcapsules in the 10–100 µm range are characterised by image analysis, either manually or using image processing software^48^. This analysis is normally limited to 50–100 particles per sample. Laser scattering methods can be unreliable in this size regime, particularly for core-shell particles, as several assumptions about density, refractive index, particle shape, and stability under measurement conditions must be made that do not generally hold for such materials^49^. We therefore decided to explore video processing as an alternative method that would allow the analysis of many more droplets per sample, taking advantage of the continuous production and inline video monitoring of droplets. Still images of droplets and microcapsule were analysed using ImageJ. Videos of droplet production were processed using custom Python code; methodology and limitations are discussed in the SI. Version 1.0 of this code is available under https://github.com/fsimkovic/droplet-assessment.

## Results and Discussion

Homogenized emulsions were found to contain droplets from 1–16 µm, with a broad, bimodal distribution of droplet sizes (Fig. 1a,c), as typical for droplets produced by this method^43^. Larger or smaller average droplet sizes can be generated by changing the stirring speed, but high polydispersity always results owing to the variable shear forces experienced in batch processes.

**Figure 2:**
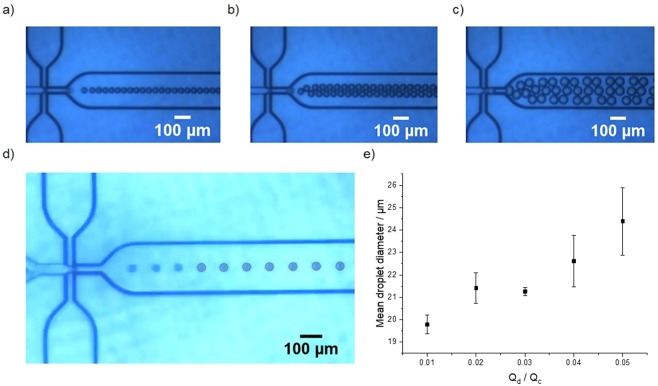
Size control of limonene droplets produced in a microfluidic chip (**a**–**c**) Optical microscope images of o/w emulsions produced at Q_c_ and Q_d_ of (**a**) Q_c_ = 100 µL min^−1^, Q_d_ = 5 µL min^−1^; (**b**) Q_c_ = 100 µL min^−1^, Q_d_ = 10 µL min^−1^; (**c**) Q_c_ = 20 µL min^−1^, Q_d_ = 5 µL min^−1^. (**d**) example of still from microscope video that has undergone processing to detect and measure droplet diameter via Python code (Q_c_ = 100 µL min^−1^, Q_d_ = 1 µL min^−1^ - see SI for full details); (**e**) plot of Q_d_/Q_c_ vs. mean emulsion droplet diameter analysed by video processing of >20000 droplets per video. Q_c_ is fixed at 100 µL min^−1^. Error bars represent standard deviation.

By contrast, droplets produced in the microfluidic chip were characterised by narrow dispersity (Fig. 1b,d). Relative flow rates that produced single streams of droplets were targeted to avoid the production of aggregated particles in the polymerisation step; however, smaller or larger droplet sizes could readily be produced by widening the range of flow rates used (Fig. 2a–c). Average droplet diameters between 20–26 µm were measured via video analysis when Q_d_ was varied from 1–5 µL min^−1^ (Fig. 2d,e); an increase in droplet diameter was observed with increased Q_d_.

**Figure 3:**
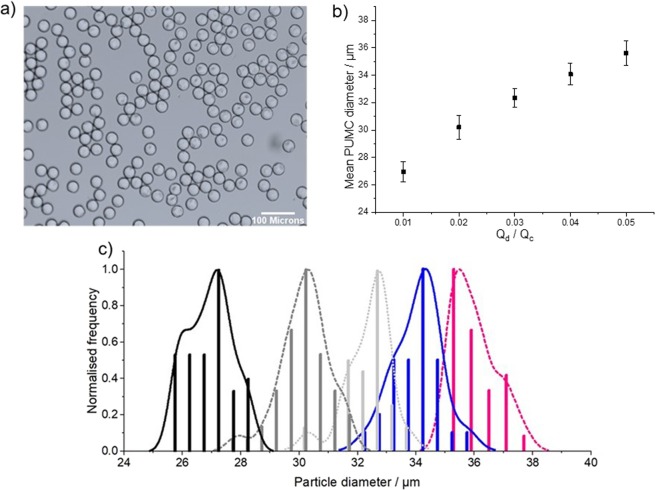
Production of size-controlled limonene microcapsules: (**a**) Optical microscope image of PUMC from an emulsion produced at Q_c_ = 100 µL min^−1^, Q_d_ = 5 µL min^−1^; (**b**) plot of Q_d_/Q_c_ vs. mean PUMC diameter analysed by image processing of ~50 capsules. Q_c_ is fixed at 100 µL min^−1^. (**c**) histogram of PUMC diameter analysed by image processing of ~50 PUMCs per optical microscope image. Q_d_ = 1 µL min^–1^ (black solid lines); 2 µL min^–1^ (dark grey dashed line); 3 µL min^–1^ (light grey dotted line); 4 µL min^–1^ (blue solid line); 5 µL min^–1^ (magenta dashed line). Q_c_ is fixed at 100 µL min^−1^. Error bars represent standard deviation.

Narrow dispersity was also observed in the microcapsules produced via subsequent polymerisation of the droplets (Fig. 3a–c). Again, we observed an increase in PUMC size with an increase of Q_d,_ allowing rapid access to ‘learning sets’ of size-controlled microcapsules. Average PUMC diameters of 27, 30, 32, 34, and 35 µm produced at Q_c_ = 100 µL min^−1^ and Q_d_ of 1, 2, 3, 4, and 5 µL min^−1^ respectively were measured by still image analysis.

Although it is tempting to compare the sizes of the droplets (20–26 µm, Fig. 2e) and the resultant PUMCs (27–35 µm Fig. 3b), these sets of results are not directly comparable due to the different image processing techniques, and conditions under which the images were obtained (through a glass chip vs. on a glass slide – see SI for detailed discussion). The clear advantage of automated video processing is in enabling the easy processing of tens of thousands of droplets; in this iteration, we sacrifice some accuracy to enable rapid processing (see SI for detailed discussion of the origin of this inaccuracy). In future, a more sophisticated approach, such as a supervised Machine Learning algorithm, could be trained to detect droplets; we anticipate this approach would greatly improve accuracy and enable high quality, automated measurement of droplet sizes as they are generated^50^.

**Figure 4:**
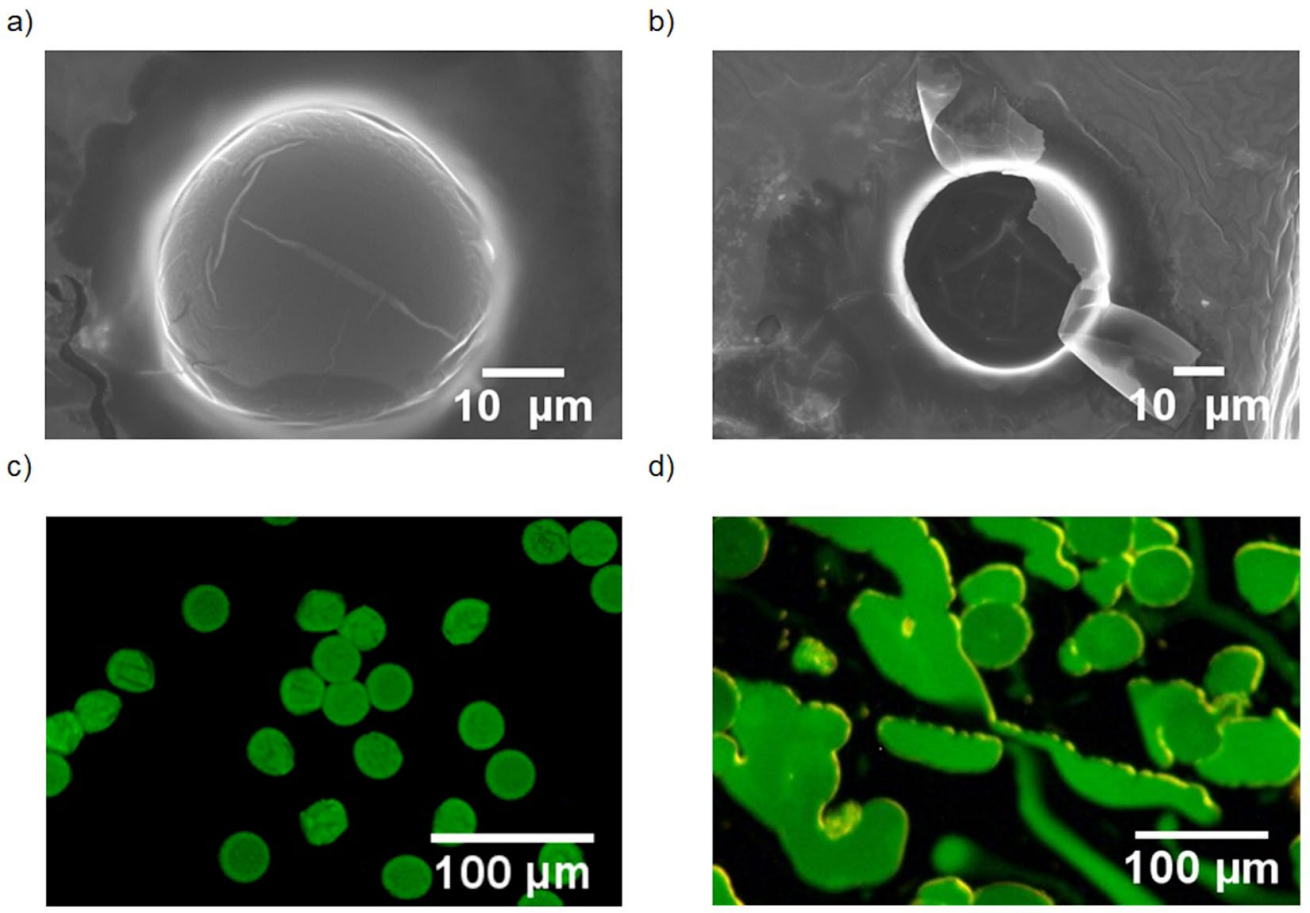
Release of limonene from PUMC: (**a**,**b**) SEM images of (**a**) intact and (**b**) burst PUMCs produced by microfluidics; (**c**,**d**) confocal fluorescence microscopy images of (**c**) intact PUMCs produced by microfluidics after 24 h drying; (**d**) the same microcapsules after application of finger-pressure onto the glass slide.

Microcapsules were characterised by SEM (Fig. 4a,b); both intact and burst particles were observed after exposure to the high vacuum conditions required for SEM imaging, confirming their hollow nature. The microcapsules were observed to have poor stability under the electron beam, eroding during extended exposure and therefore making it difficult to accurately assess shell thickness. From the images obtained, we estimate a shell thickness of ~100 nm.

To visualise the liquid core of the microcapsules, an emulsion containing fluorescent dye (Hostasol yellow 3 G) was generated in the microfluidic chip and subjected to encapsulation via IFP using the protocols described above. The resultant microcapsules were dried on a glass slide for 24 h before imaging with confocal fluorescence microscopy (Fig. 4c). To release the fluorescent payload, a gentle pressure was then applied via a second glass slide (Fig. 4d), indicating that these microcapsules may have utility in applications where pressure-sensitive release is desirable – for example, fragrance release in deodorants.

## Conclusions

A series of monodisperse polymer microcapsules was produced using microfluidic methods and using sustainable materials. By using video processing to analyse the size distributions of the droplets produced, we can rapidly and automatically establish narrow dispersity, and measure changing droplet size when using different flow rates. Such straightforward and adaptable methodologies are readily extendable to other chemistries, different particle sizes, and new payloads for diverse applications. It has been previously demonstrated that shell thickness and permeability can be tuned by careful choice of surfactant and polymerisation chemistry^24,38–40^. By exploiting this tunability in combination with the size control demonstrated in this work, we anticipate the production of bespoke sustainable microcapsules for commercial formulations, healthcare, and energy and sustainability applications. Such control over microcapsule production will enable new applications and products as well as facilitating greater understanding of the impact of particle size on function.

### Supporting data statement

Details of image and video processing methods are available in the Supporting Information. Please contact the corresponding author regarding requests for data. Version 1.0 of the source code used in this study is available under https://github.com/fsimkovic/droplet-assessment.

## Supplementary information

Supporting information for Production of monodisperse polyurea microcapsules using microfluidics
