# Supplementary material for: Production of monodisperse polyurea microcapsules using microfluidics
Source: Sci Rep. 2019 Nov 29;9:17983. doi: 10.1038/s41598-019-54512-4 (PMC6884639; doi:10.1038/s41598-019-54512-4)
Supplement: Supplementary file 1 — Supporting information for Production of monodisperse polyurea microcapsules using microfluidics [file 41598_2019_54512_MOESM1_ESM.docx]

**Supporting Information**

**Production of monodisperse polyurea microcapsules using microfluidics**

*Michael F. Thorne,^1^ Felix Simkovic,^2^ and Anna G. Slater^,1,*^*

^1^ Department of Chemistry and Materials Innovation Factory, University of Liverpool, Crown Street, Liverpool, L69 7ZD; ^2^Institute of Integrative Biology, University of Liverpool, Liverpool, L69 7ZB

*To whom correspondence should be addressed. Email [anna.slater@liverpool.ac.uk](mailto:anna.slater@liverpool.ac.uk)

**1. Experimental section**

**(**R)-(+)-Limonene (97%) was purchased from Alpha Aesar. Hostasol Yellow 3G was purchased from Clariant. All other materials were purchased from Sigma Aldrich without further purification.

**1.0 Microfluidic chip**

Glass chips were purchased from Dolomite Microfluidics and used as received. A hydrophilic 2 reagent droplet chip with a 50 µm etch depth was used for droplet production. Different sized emulsions can be readily achieved by altering flow rates or using alternative chip geometries and channel depths.

**1.1 Droplet generation system**

A droplet generation system was purchased from Dolomite Microfluidics, and briefly comprised of: 2 Mitos pressure pumps equipped with flow rate sensors and pressurized using compressed air; oil and water delivery lines equipped with inline filters and isolating taps; a ‘Meros’ high speed digital microscope and stage.

**1.2 Microfluidic emulsion droplet and microcapsule formation and optical microscopy**

The microfluidic chip junction was imaged in operation using the inline high speed microscope supplied with the Dolomite system (**Fig S1**), via which still images and videos were obtained. The dispersed phase, MDI in (R)-(+)-Limonene (0.3 wt.%) was flowed through the central channel, and the continuous phase, an aqueous solution of SDS (1.0 wt.%) and NaCl (1.5 wt.%), was flowed through the top and bottom channels. Droplets of different diameters were produced by varying the flow rate of dispersed phase, Q_d_, while maintaining the flow rate of the continuous phase, Q_c_ = 100 µL∙min^–1^. All solutions were filtered through a 0.45 µm pore size syringe filter prior to use to prevent chip blockages. The emulsions obtained were collected at the chip exit and imaged using offline optical microscopy (**Fig S2**) with an Olympus BX53 optical microscope equipped with an Olympus DP26 digital colour camera and Stream image analysis software.


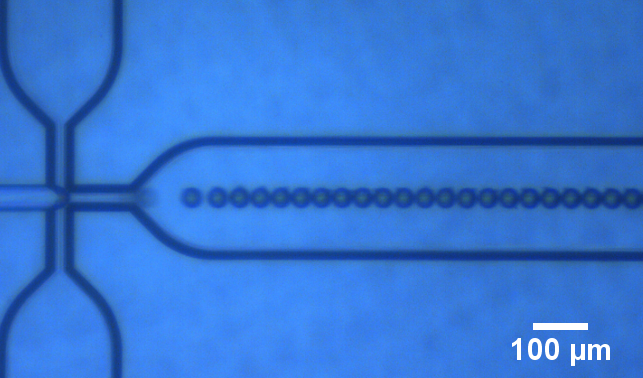


**Figure S1.** Formation of monodisperse O/W droplets with Q_d_ = 5 µL∙min^–1^


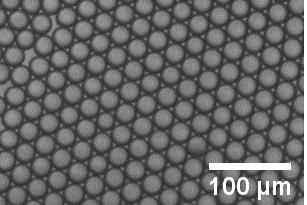


**Figure S2.** O/W droplets formed at Q_d_ = 5 µL∙min and imaged on a glass slide using offline optical microscopy

**Scheme S1.** Reaction scheme of urea formation from TEPA and MDI.

To obtain polyurea microcapsules (**Scheme S1**), the chip output was collected into a solution of TEPA (3.0 wt.%), SDS (1.0 wt.%) and NaCl (1.5 wt.%) in distilled water (10 mL). During the 15 minute collection, the droplet suspension was gently stirred at 100 RPM at room temperature; the solution was then left unstirred for 24 h to ensure full capsule formation. The microcapsules obtained were imaged using offline optical microscopy (**Fig S3**) as above.


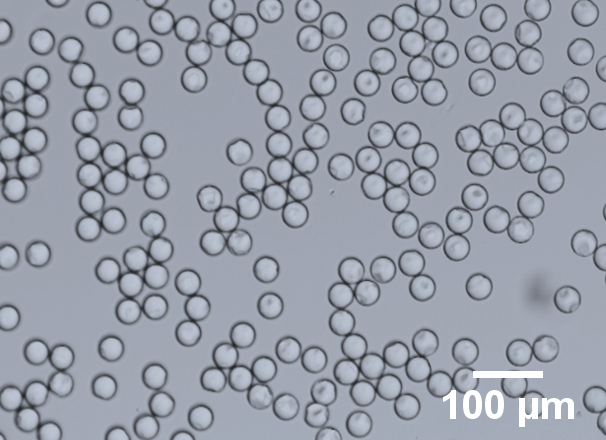


**Figure S3:** PUMCs produced at Q_d_ = 5 µL∙min^–1^ and imaged on a glass slide using offline optical microscopy.

**1.3 Homogeniser emulsion droplet and microcapsule formation**

A solution of SDS (1.0 wt.%) and NaCl (1.5 wt.%) in distilled water (200 ml) was prepared, followed by a solution of MDI (0.3 wt.%) in (R)-(+)-Limonene (10 mL). These solutions were homogenised at 8000 RPM for 2 minutes using an ULTRA-TURRAX T-25 homogeniser. To form microcapsules, an aliquot of the emulsion (1 ml) was injected into of a solution of TEPA (3.0 wt.%), SDS (1.0 wt.%) and NaCl (1.5 wt.%) in distilled water (10 ml). The droplet suspension was gently stirred at 100 RPM at room temperature for 15 minutes with a magnetic flea then left undisturbed for 24 h to ensure full capsule formation before creaming.

**2. Size distribution analysis**

**2.1 Still image processing**

Optical images were processed using ImageJ software and the ‘WandAutoMeasure Macro’ tool available within the software, which allows the user to select microcapsules and generate their area. Still images were first converted to their monochrome equivalent (**Fig S4**). Microcapsule diameters were then calculated from the area data. At least 50 particles or emulsion droplets were used to analyse size distribution.


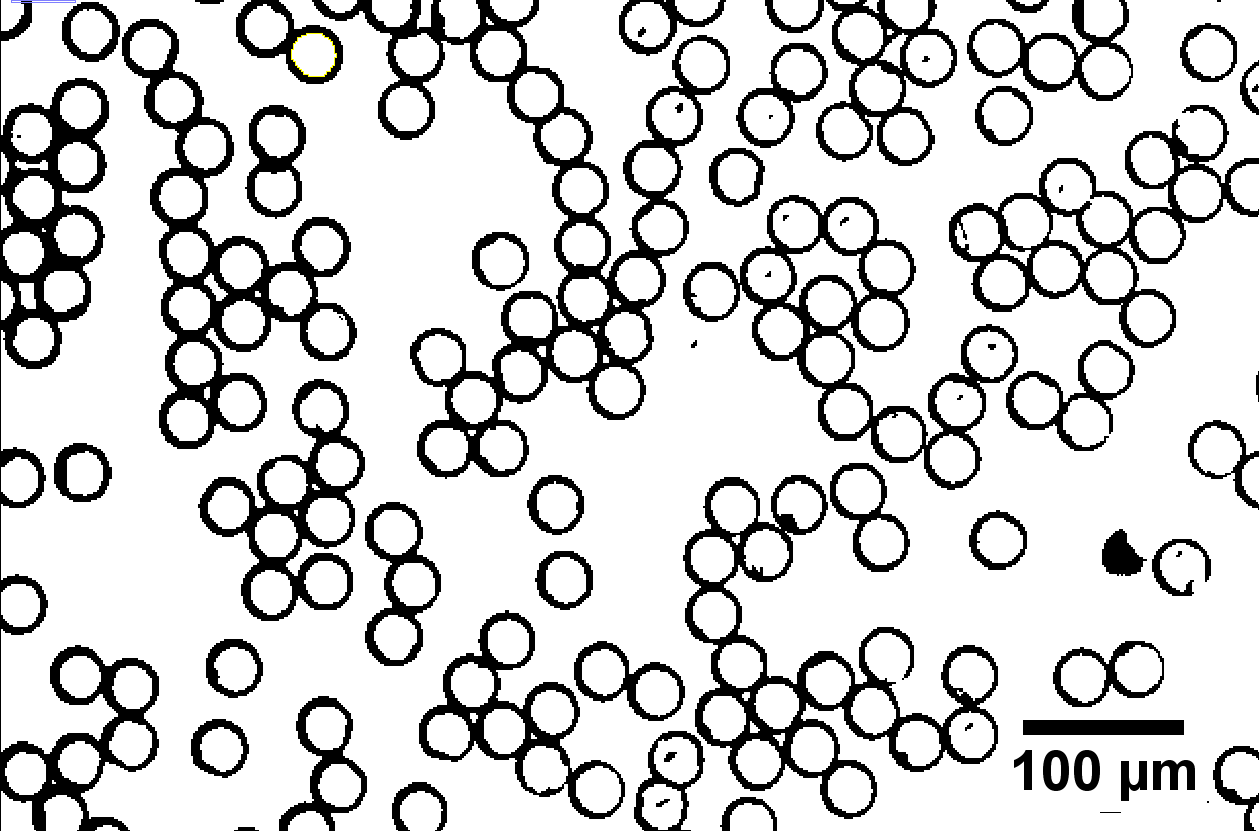


**Figure S4:** PUMCs produced at Q_d_ = 5 µL min^–1^ with dark circles indicating particles used for particle size distribution analysis by ImageJ.

**2.2 Video processing**


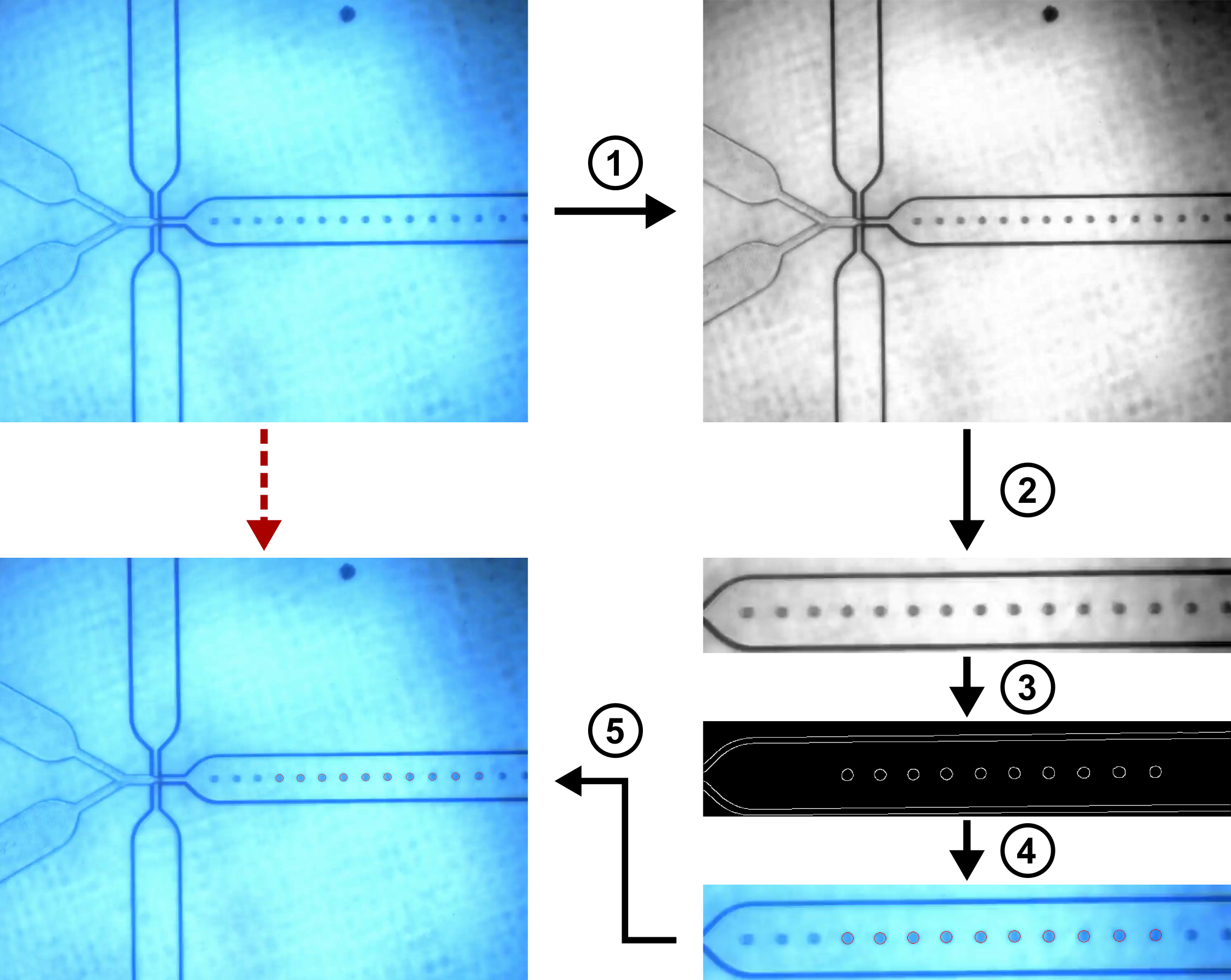


**Figure S5**: Processing of sample frame for droplet detection in video footage of droplets produced at Qd = 1 µL min^–1^. The dashed red line highlights the desired process. (1) Each frame is converted to its grayscale equivalent. (2) A subframe containing the droplets is extracted, and (3) a Canny filter is applied for edge detection. (4) Hough transform was used to detect hough peaks in the edge-detected subframe, and the radii of the 10 most dominant peaks measured. (5) In the last step, the identified peaks are fitted back to the original full frame. Scikit-image (v0.13.1)^1^ was used to perform all steps. Version 1.0 of the source code used in this study is available under <https://github.com/fsimkovic/droplet-assessment>.

**2.3 Discussion of relative merits of image and video processing**

Both methods of particle size imaging used in this paper are based on measuring numbers of pixels within droplets, and both require the image to be processed so that droplet edges have higher contrast and can be detected. Thus, error is readily introduced as a consequence of the choices of the operator (e.g. the thickness of the circle generated by the ImageJ macro used to define the edges of the droplet has an effect on a) the area measured and b) the number of droplets that are successfully identified as such; a balance is struck so that both parameters are at acceptable levels, but again this is a choice of the operator). Furthermore, the conditions under which the image is obtained will have an effect on the apparent size of the droplet – for example, the droplets in the chip are surrounded by water and glass, whereas there is a thin film of water over the droplets on a glass slide.

With these caveats in mind, however, it is possible to draw conclusions regarding droplet size in different videos provided the videos are obtained and treated in the same way; a clear advantage of using an automated method is that no operator decisions need to be made, reducing the opportunity for error. As tens of thousands of droplets can be measured without the need for tedious manual image processing, we anticipate that such methods will be useful as an ‘in process check’, ensuring that droplet size does not drift over time – important for scale up.

One advantage of manual image processing is that errors in droplet identification can be easily spotted and excluded; this becomes very challenging or even impossible when the numbers of droplets analysed increases to >20000. In the data reported in the paper, the number of ‘false droplets’ counted by the code was less than 10 % in all cases, but was a more pronounced problem when droplets were produced in close proximity to each other at higher rates of Q_d_; we hypothesise that this contributes to the larger error bars at higher flow rates (**Fig 2e** in the main paper). The use of a more sophisticated approach, such as a supervised Machine Learning algorithm that could be trained to detect droplets, would potentially reduce this problem.

1. S. van der Walt, J. L. Schonberger, J. Nunez-Iglesias, F. Boulogne, J. D. Warner, N. Yager, E. Gouillart, T. Yu and S. I. Contributors, *Peerj*, 2014, **2**.
